# Surface Plasmon Enhancement of an InGaN Quantum Well Using Nanoparticles Made of Different Metals and Their Combinations

**DOI:** 10.3390/nano12030370

**Published:** 2022-01-24

**Authors:** Muhammad Farooq Saleem, Yi Peng, Liuyan Li, Bangdi Zhou, Jia Yang, Haixia Lu, Guoxin Li, Lixiang Huang, Jie Chen, Wenwang Wei, Yanlian Yang, Yukun Wang, Wenhong Sun

**Affiliations:** 1 Research Center for Optoelectronic Materials and Devices, School of Physical Science & Technology, Guangxi University, Nanning 530004, China; farooq@mail.ustc.edu.cn (M.F.S.); 1907401031@st.gxu.edu.cn (Y.P.); 2007301071@st.gxu.edu.cn (L.L.); 1907301114@st.gxu.edu.cn (B.Z.); 2007301168@st.gxu.edu.cn (J.Y.); heathler2022@outlook.com (H.L.); 13929711637@163.com (G.L.); hlx18277136091@outlook.com (L.H.); a709161389@outlook.com (J.C.); 1814404039@st.gxu.edu.cn (W.W.); kindy789456@126.com (Y.Y.); jiaaikun@126.com (Y.W.); 2 Guangxi Key Laboratory for Relativistic Astrophysics, School of Physical Science & Technology, Guangxi University, Nanning 530004, China; 3 Guangxi Key Laboratory of Processing for Non-Ferrous Metallic and Featured Materials, Guangxi University, Nanning 530004, China

**Keywords:** light-emitting diodes, surface plasmon, nanoparticles, photoluminescence

## Abstract

Surface plasmon (SP) enhancement of photoluminescence (PL) from a green-emitting InGaN/GaN quantum well (QW) using nanoparticles (NPs) made of different metals and their combinations was investigated. The NPs were formed by annealing the metal films in N_2_ followed by rapid cooling. Four-fold enhancement in PL intensity was achieved using random metal NPs made of Cu on Mg (Cu-Mg) double metal film that was more than two folds of the enhancement observed by AgNPs. Reversing the order of metal film deposition (Mg on Cu) resulted in much lower PL intensity due to significantly different NPs size distribution as the given annealing conditions did not cause homogeneous alloying of the two metals. The results pave the way for the application of NPs of relatively low-cost unconventional metals and their combinations in the SP enhancement of LEDs.

## 1. Introduction

One of the most important applications of LEDs is white light production by mixing the lights of green, blue, and red colors, which is currently limited by the low external quantum efficiency (EQE) of green LEDs known as the “green gap” [1,2]. III-nitride semiconductor-based QW-LEDs are used for this purpose to allow the tuning of emission from visible to UV simply by controlling the composition ratio in the QW active region [3,4]. Green LEDs are realized by increasing In concentration in InGaN QWs. The increased In concentration results in an obvious decline of material quality that leads to poor efficiencies for LEDs performing in longer wavelength ranges [5,6]. In the last two decades, SP enhancement of light emission in LEDs has been investigated as one of the most promising methods [3,7,8]. Thin films, NPs, and gratings made of Au, Ag, and Al have been employed to excite SPs in LEDs [9]. In QW-LEDs, the light produced as a result of exciton recombination in QW can couple with the electromagnetic field produced by the SPs in a metal, creating an additional recombination channel [10]. This additional recombination channel results in increased radiative recombination efficiency [10,11,12].

NPs made by annealing the multilayers of different metals have been reported to allow tuning of the SP energies to resonate with emission energies of QWs [13]. There is also a possibility of alloying of the two or more metals depending on the annealing conditions, which might increase the shelf life of NPs due to the well-known oxidation resistance of some alloys. Moreover, the use of precious metals for SP enhancement application in LEDs increases the overall fabrication cost. It is of profound importance to explore the plasmonic capabilities of cost-effective materials for such applications. Some of the metals or their combinations might have the ability to outperform the conventional Ag, Au, or Al metals. The dispersion relations for Cu and Mg metals, for instance, point towards their promising plasmonic properties, which should be explored further [14,15]. The NPs made by combining the two or more such metals may offer further control of the tuning of their SP resonance. In this study, we explored the possibilities of further enhancement of LED emission by using NPs made of relatively cheaper and unconventional metals and their combinations.

## 2. Materials and Methods

**Figure 1:**
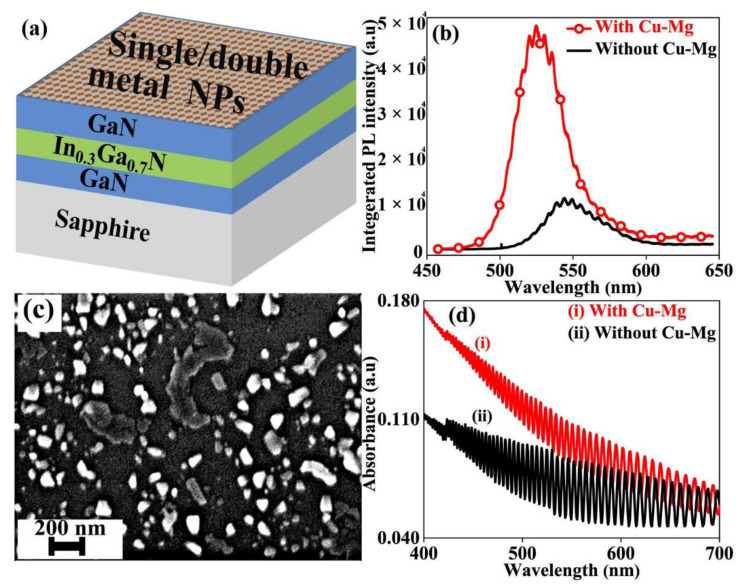
(**a**) Schematic representation of the structure of SP-enhanced QW used in this study. (**b**) Comparison of room-temperature PL spectra of reference sample with that of Cu-Mg sample. (**c**) SEM image of the Cu-Mg NPs. (**d**) Comparison of UV-visible absorption of the reference and Cu-Mg NPs sample.

The reference sample is a typical green-LED epitaxial wafer that contains a 2 nm thick In_0.3_Ga_0.7_N single-QW structure sandwiched between two GaN layers grown on a double-sided polished sapphire substrate using MOCVD, as shown in the schematic diagram in Figure 1a. The top GaN layer is kept only 10 nm thick to allow strong QW-SP coupling. The bottom GaN layer is 10 μm thick. The wafer was cut into pieces and various metals, and their combinations (10 nm each) were deposited on the top GaN surface using the thermal evaporation method. The metals used included Ag, Al, Cu, Mg,(Hebei Zhongyue Metal Material Technology Co., Ltd., Shijiazhuang, Hebei, China) and their combinations. NPs were formed by annealing single- and double-metal films in N_2_ at 300 °C in a quartz tube furnace filled with N_2_. The quartz tube was pumped for 20 min before N_2_ was filled in it. A 457 nm laser of Zolix Finder One Raman Microscopic Spectrograph (Zolix Instruments Co., Ltd., Beijing, China) was used for PL measurement from the polished sapphire side. The reference sample was also annealed at 300 °C in N2 to balance annealing effects for a true comparison of PL. The surface image was taken using the JEOL JEM5610 scanning electron microscope (SEM) (JEOL, Ltd., Tokyo, Japan). A double-beam ultraviolet–visible spectrophotometer TU1901 (Beijing Purkinje General Instrument Co., Ltd., Beijing, China) was used to measure UV–visible absorption and transmission spectra.

## 3. Results and Discussion

SP enhancement is found to be more effective for LEDs with intrinsically poor internal quantum efficiency (IQE) and relatively small GaN thicknesses [16,17]. It has also been observed that the topmost QW contributes the most to SP enhancement in a multiple-QW system [18]. The reference sample used in our study with single QW and 10 nm top GaN thus is a perfect platform for the investigation of SP enhancement of PL.

Among the samples with NPs of various metals and their combinations, NPs made of a Cu-Mg combination resulted in highest PL enhancement. Here, Cu-Mg NPs refer to those formed by annealing the 10 nm Cu on 10 nm Mg film stack. Figure 1b shows the comparison of the PL spectrum of the reference sample (without metal NPs on the top) with that of the Cu-Mg sample. The observed PL peak of the Cu-Mg sample is four times higher than that of the reference sample. The PL peak associated with the reference sample was centered at 548 nm blue shifts to 520 nm with Cu-Mg NPs. The peak position is mainly defined by the QW emission wavelength and is influenced by the position of SP resonance wavelength. The blue shift with Cu-Mg NPs is attributed to the stronger SP coupling on the high-energy side due to the higher density of states of SPs in the shorter-wavelength range [10]. Temperature dependent PL (TDPL) was used to calculate the IQE values (Figure S1). The single-QW sample had an IQE as low as 2.2%, which increased to 8% with Cu-Mg NPs.

Cu-Mg NPs of random shapes and sizes are formed as shown in the SEM image in Figure 1c. The comparison of the UV-visible absorption spectrum of the Cu-Mg sample with that of the reference sample is shown in Figure 1d. The absorption increases below 700 nm for the Cu-Mg sample with peak absorption somewhere below 400 nm. Further improvement in enhancement can be achieved by controlling the geometry and size distribution of NPs to match the absorption peak of Cu-Mg NPs with QW emission wavelength for stronger resonance. A strong well-defined absorption peak in resonance with the emission wavelength is usually taken as a guarantee for strong SP–QW coupling [13,19]. This is, however, controversial since higher SP enhancement has also been observed by NPs of extremely weak absorption compared with NPs of high absorption in the desired QW emission wavelength range [20,21]. It has been found that SP enhancement is rather a combined effect of absorption, scattering, scattering-to-extinction ratio, and field enhancement [3,22]. SP enhancement in LEDs depends on many factors such as metal type, geometry and size distribution of NPs, QW period number, QW emission wavelength, and GaN thickness [23].

**Figure 2:**
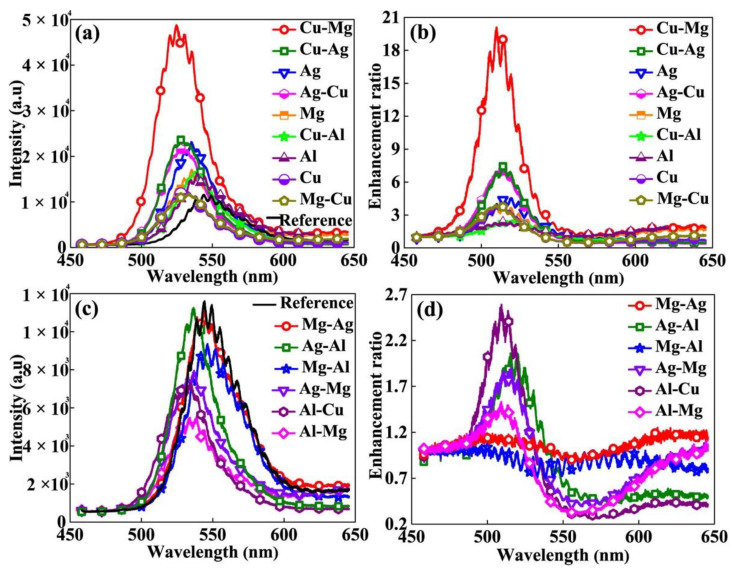
(**a**) Comparison of PL spectra of metal NPs containing samples that resulted in normalized intensities higher than that of the reference sample. (**b**) PL intensity ratios calculated from the PL data shown in (**a**). (**c**) Comparison of PL spectra of metal NPs containing samples that resulted in normalized intensities comparable to and lower than reference sample. (**d**) PL intensity ratios calculated from the PL data shown in (**a**).

Figure 2a shows the comparison of normalized PL spectra of the structures with various metals and their combinations on the top GaN surface. The PL intensity of the Cu-Mg structure is highest among the structures used in this study at a level that is more than twice the intensity of the most widely used Ag metal-containing structure. The second highest PL intensity is observed by the Cu-Ag structure and also resulted in PL intensity higher than that of AgNPs structure. It should be noted that PL blue-shifted with most of the metals on the top, which indicates that the SP resonance energies lie in the shorter-wavelength ranges. Enhancement can be improved by controlling the metal NPs geometries and size distribution, optimizing the top GaN thickness and QW period number and by inserting a dielectric interlayer between metal NPs and GaN [17,24].

Figure 2b shows the comparison of enhancement ratios that are estimated by dividing the PL intensities of metal-containing structures with those of the reference sample. The enhancement occurs exclusively in the 470–560 nm wavelength range for all the samples as the characteristics emission peak of QW lies in this range. The Cu-Mg structure results in more than four folds of the enhancement observed by Ag-containing structure. The second highest enhancement ratio is observed for Cu-Ag and Ag-Cu NPs. Interestingly, the Mg-Cu (reversing the order of metal deposition) did not result in significant PL enhancement. This confirms that the given annealing conditions did not support homogenous alloying of Cu and Mg. Reversing the order of Cu and Ag film deposition on the other hand resulted in comparable enhancement ratios, which might be because of the interdiffusion of the two metals under the same annealing conditions. Interestingly, Mg, which is reported to be a good candidate for the near-UV wavelength range, also gives considerable enhancement in this study [25].

SP enhancements are affected by the extent of SP resonance with the excitation laser and the QW emission wavelength. The light of relatively shorter wavelengths cannot resonate well with the SP modes of long wavelengths [26]. With 457 nm laser and 520 nm emission from the QW structure used in this study, most of the SPs from different metals should be in resonance with both the laser and the emission. This should in principle result in higher PL enhancements compared with those reported by 400 and 405 excitation lasers. K. Tateishi et al., for instance, have reported a giant enhancement ratio using an Al film that did not result in significant enhancement in our work due mainly to the unique nanoscale surface properties of the Al film used in their work, which resulted in efficient SP enhancement [27]. The nanoscale surface properties such as roughness and texture are, however, not easy to control. Different metal deposition methods and treatments can result in different surface properties.

Figure 2c shows the PL spectra for metals and their combinations that did not result in a significant increase in PL intensities. The optimization of geometries and size distribution of these NPs and the LED structures might still allow enhancements from these metals. Figure 2d shows the enhancement ratios estimated from the PL spectra shown in Figure 2c.

**Figure 3:**
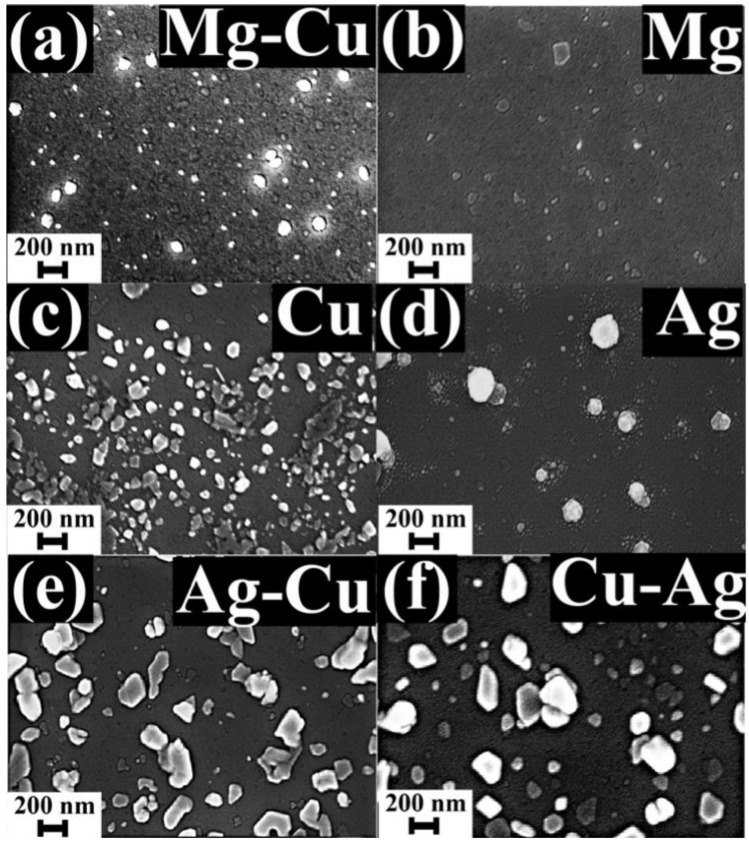
Surface images of NPs made of (**a**) Mg on Cu, (**b**) Mg, (**c**) Cu, (**d**) Ag, (**e**) Ag on Cu, and (**f**) Cu on Ag metals.

Figure 3 shows the SEM surface images of some metal NPs. Compared with Cu-Mg NPs shown in Figure 1c, the Mg-Cu NPs are smaller in size, and the surface coverage of the NPs also drops when the Mg is on the top of Cu. It can be concluded that the order of metal deposition (Mg on Cu vs. Cu on Mg) affects the size distribution of the NPs, leading to differences in the extent of SP enhancement. It should be noted that Mg on top of most of the metals resulted in poor enhancement. Under the given annealing conditions, Mg did not form NPs as shown in Figure 3b, which indicates that the NPs shown in Figure 1c are CuNPs on Mg film.

The Cu NPs shown in Figure 3c do not look much different from the Cu-Mg NPs, yet the enhancement from Cu NPs is much lower compared with Cu-Mg NPs. Figure 3d shows the big Ag NPs surrounded by small Ag NPs. The small-Ag NPs surrounding the big NPs have been reported to result in PL quenching [28]. Cu-Ag and Ag-Cu double metals films on the other hand resulted in similar NPs size and surface coverage, resulting in almost equal PL enhancement. The size distribution of NPs is thus an important factor that can be further optimized by using different annealing temperatures and the thicknesses of the metals involved. Cu in combination with Ag and Mg thus resulted in superior SP properties. The SP capabilities of Cu in combination with other metals should hence be explored further.

## 4. Conclusions

SP enhancement capabilities of single- and double-metal NPs for a green-emitting QW were investigated. Cu-Mg NPs resulted in high PL and IQE enhancement compared with that of NPs of all other metals and their combinations studied in this work. Reversing the order of metal deposition (Mg-Cu) resulted in low PL intensity, which confirmed that the homogenous alloying of Cu and Mg did not occur under the given annealing conditions. The order of metal deposition can affect the geometry and size distribution of the NPs made of some metal combinations, leading to their different SP enhancement capabilities. It has been observed that, under the given annealing conditions, Mg film does not form NPs and also limits the NPs formation in a metal under it. A better control of the geometry and size distribution may offer further emission enhancement and understanding of the mechanism of emission enhancement using Cu-Mg NPs that will be explored in future work. The work emphasizes the use of NPs of low-cost metals and their combinations for SP enhancement in LEDs.

## Data Availability

The data presented in this study are available on request from the corresponding author.

## Supplementary Materials

The following supporting information can be downloaded at: https://www.mdpi.com/article/10.3390/nano12030370/s1, Figure S1. Room temperature PL compared with PL obtained at 20 K for the sample (**a**) without metal and the sample (**b**) with Cu-Mg NPs taken by 457 nm laser.
