# Supplementary material for: Surface Plasmon Enhancement of an InGaN Quantum Well Using Nanoparticles Made of Different Metals and Their Combinations
Source: Nanomaterials (Basel). 2022 Jan 24;12(3):370. doi: 10.3390/nano12030370 (PMC8839922; doi:10.3390/nano12030370)
Supplement: Supplementary file 1 [file nanomaterials-12-00370-s001.zip › nanomaterials-1525609-supplementary.pdf]

nanomaterials

Supplementary Information

Surface Plasmon Enhancement of an InGaN Quantum Well Using Nanoparticles Made of Different Metals and Their Combinations

Muhammad Farooq Saleem [1], Yi Peng [1], Liuyan Li [1], Bangdi Zhou [1], Jia Yang [1], Haixia Lu [1], Guoxin Li [1], Lixiang Huang [1], Jie Chen [1], Wenwang Wei [1], Yanlian Yang [1], Yukun Wang [1] and Wenhong Sun [1,2,3,*]

[1] Research Center for Optoelectronic Materials and Devices, School of Physical Science & Technology, Guangxi University, Nanning 530004, China; farooq@mail.ustc.edu.cn (M.F.S.); 1907401031@st.gxu.edu.cn (Y.P.); 2007301071@st.gxu.edu.cn (L.L.); 1907301114@st.gxu.edu.cn (B.Z.); 2007301168@st.gxu.edu.cn (J.Y.); heathler2022@outlook.com (H.L.); 13929711637@163.com (G.L.); hlx18277136091@outlook.com (L.H.); a709161389@outlook.com (J.C.); 1814404039@st.gxu.edu.cn (W.W.); kindy789456@126.com (Y.Y.); jiaaikun@126.com (Y.W.)

[2] Guangxi Key Laboratory for Relativistic Astrophysics, School of Physical Science & Technology, Guangxi University, Nanning 530004, China

[3] Guangxi Key Laboratory of Processing for Non-Ferrous Metallic and Featured Materials, Guangxi University, Nanning 530004, China

* Correspondence: youzi7002@gxu.edu.cn

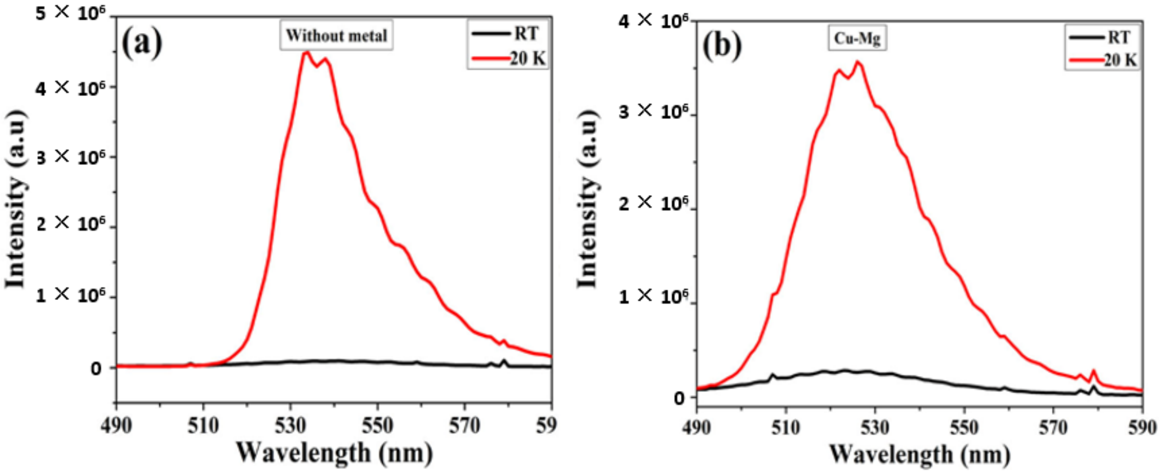

Figure S1. Room temperature PL compared with PL obtained at 20 K for the sample (**a**) without metal and the sample (**b**) with Cu-Mg NPs taken by 457 nm laser.
